# The efficacy of a multimodal physical activity intervention with supervised exercises, health coaching and an activity monitor on physical activity levels of patients with chronic, nonspecific low back pain (Physical Activity for Back Pain (PAyBACK) trial): study protocol for a randomised controlled trial

**DOI:** 10.1186/s13063-017-2436-z

**Published:** 2018-01-15

**Authors:** Crystian B. Oliveira, Márcia R. Franco, Chris G. Maher, Anne Tiedemann, Fernanda G. Silva, Tatiana M. Damato, Michael K. Nicholas, Diego G. D. Christofaro, Rafael Z. Pinto

**Affiliations:** 1 Department of Physical Therapy, Faculty of Science and Technology, Sao Paulo State University (UNESP), Presidente Prudente, SP Brazil; 2 School of Public Health, Sydney Medical School, University of Sydney, Sydney, NSW Australia; 3 Institute for Musculoskeletal Health, Sydney Local Health District, Sydney, NSW Australia; 4 Pain Management Research Institute, University of Sydney at Royal North Shore Hospital, Sydney, NSW Australia; 5 Departament of Physical Education, Faculty of Science and Technology, Sao Paulo State University (UNESP), Presidente Prudente, SP Brazil; 6 Departament of Physical Therapy, Universidade Federal de Minas Gerais (UFMG), Belo Horizonte, MG Brazil

**Keywords:** Low back pain, Physical activity, Exercise therapy, Health coaching, Activity monitor

## Abstract

**Background:**

Physical activity plays an important role in the management of chronic low back pain (LBP). Engaging in an active lifestyle is associated with a better prognosis. Nevertheless, there is evidence to suggest that patients with chronic LBP are less likely to meet recommended physical activity levels. Furthermore, while exercise therapy has been endorsed by recent clinical practice guidelines, evidence from systematic reviews suggests that its effect on pain and disability are at best moderate and not sustained over time. A limitation of current exercises programmes for chronic LBP is that these programmes are not designed to change patients’ behaviour toward an active lifestyle. Therefore, we will investigate the short- and long-term efficacy of a multimodal intervention, consisting of supervised exercises, health coaching and use of an activity monitor (i.e. *Fitbit Flex*) compared to supervised exercises plus sham coaching and a sham activity monitor on physical activity levels, pain intensity and disability, in patients with chronic, nonspecific LBP.

**Methods:**

This study will be a two-group, single-blind, randomised controlled trial. One hundred and sixty adults with chronic, nonspecific LBP will be recruited. Participants allocated to both groups will receive a group exercise programme. In addition, the intervention group will receive health coaching sessions (i.e. assisting the participants to achieve their physical activity goals) and an activity monitor (i.e. *Fitbit Flex*). The participants allocated to the control group will receive sham health coaching (i.e. encouraged to talk about their LBP or other problems, but without any therapeutic advice from the physiotherapist) and a sham activity monitor. Outcome measures will be assessed at baseline and at 3, 6 and 12 months post randomisation. The primary outcomes will be physical activity, measured objectively with an accelerometer, as well as pain intensity and disability at 3 months post randomisation. Secondary outcomes will be physical activity, pain intensity and disability at 6 and 12 months post randomisation as well as other self-report measures of physical activity and sedentary behaviour, depression, quality of life, pain self-efficacy and weight-related outcomes at 3, 6, and 12 months post randomisation.

**Discussion:**

This study is significant as it will be the first study to investigate whether a multimodal intervention designed to increase physical activity levels reduces pain and disability, and increases physical activity levels compared to a control intervention in patients with chronic LBP.

**Trial registration:**

ClinicalTrials.gov, ID: NCT03200509. Registered on 28 June 2017.

**Electronic supplementary material:**

The online version of this article 10.1186/s13063-017-2436-z) contains supplementary material, which is available to authorized users.

## Background

Low back pain (LBP) is the leading cause of years lived with disability worldwide [1], with an estimated point prevalence of 18.3% [2]. Nonspecific LBP is the most common form of LBP and the term ‘nonspecific’ means that the pathoanatomical cause is unknown [3]. Although most patients with acute LBP find that their symptoms improve in the first 6 weeks [4], about 40% of them may develop chronic and persistent symptoms [5]. Nearly 60% of these patients continue to report moderate levels of pain and disability after 1 year [6]. Emerging evidence suggests the coexistence of chronic LBP and cardiovascular comorbidities such as obesity [7] and cardiovascular diseases [8]. Hence, health care professionals are now facing the challenge of using evidence-based interventions that also manage and prevent comorbidities in this population such as interventions that aim to increase physical activity levels [9].

Physical inactivity is commonly associated with chronic LBP. There is evidence to suggest that patients with chronic LBP are less likely to meet the recommended physical activity levels [10] and are considered less active compared to individuals without LBP [11]. In addition, active patients with LBP have a better prognosis compared to sedentary patients [12]. Although exercise therapy has been endorsed by recent clinical practice guidelines [13], its effect on pain and disability are at best moderate and smaller over time [14]. A systematic review from our group shows that most physical activity-based interventions failed to increase objectively measured physical activity levels of patients with chronic musculoskeletal pain [15]. We would argue that current interventions are not designed to change patients’ behaviour toward an active lifestyle.

Behaviour-change interventions involve a range of techniques and have been advocated to change physical activity behaviour [16]. Health coaching is a behaviour-change strategy defined as the practice of health education and health promotion within a coaching context, to enhance the well-being of individuals and to facilitate the achievement of their health-related goals [17]. This strategy has been considered effective to promote physical activity among the general population [18] and among patients with acute and subacute LBP [19]. Moreover, one technique commonly incorporated in interventions to promote physical activity is the provision of feedback. Recently, wearable physical activity monitors, such as the *Fitbit*, have been used to provide interactive feedback and individualised support on real-time physical activity behaviour (e.g. step counts). Interventions incorporating wearable devices to provide feedback on physical activity have been shown to be effective in promoting weight loss among obese participants [20] as well as for increasing physical activity levels in patients with musculoskeletal pain [21].

Therefore, we will investigate the efficacy of a multimodal physical activity intervention consisting of supervised exercises, health coaching and provision of an activity monitor on physical activity levels, pain intensity and disability compared to supervised exercises plus sham coaching and sham activity monitor in patients with chronic, nonspecific LBP. Our primary hypothesis is that the physical activity intervention will increase physical activity levels as well as reduce pain intensity and disability at 3 months post randomisation. The secondary outcomes are physical activity, pain intensity and disability at 6 months and 12 months post randomisation as well as other objective measures of physical activity (i.e. time spent doing light and moderate-vigorous physical activity, number of steps), self-reported physical activity levels, depression, pain self-efficacy, perceived recovery, weight-related outcomes and quality of life measured at 3-, 6- and 12-month follow-ups.

## Methods

### Design

**Fig. 1:**
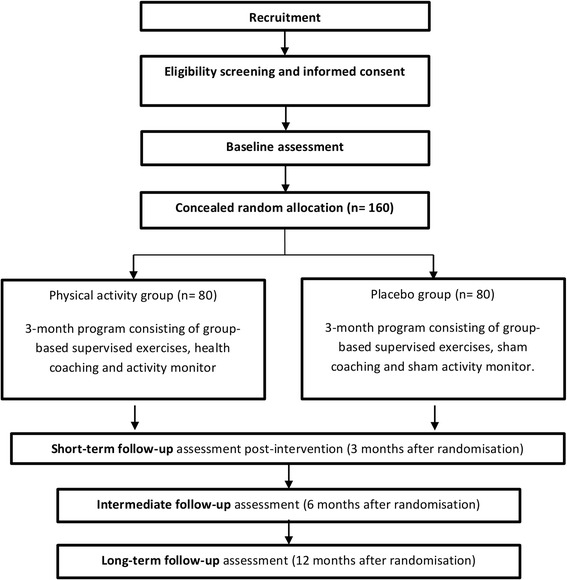
Study design

**Fig. 2:**
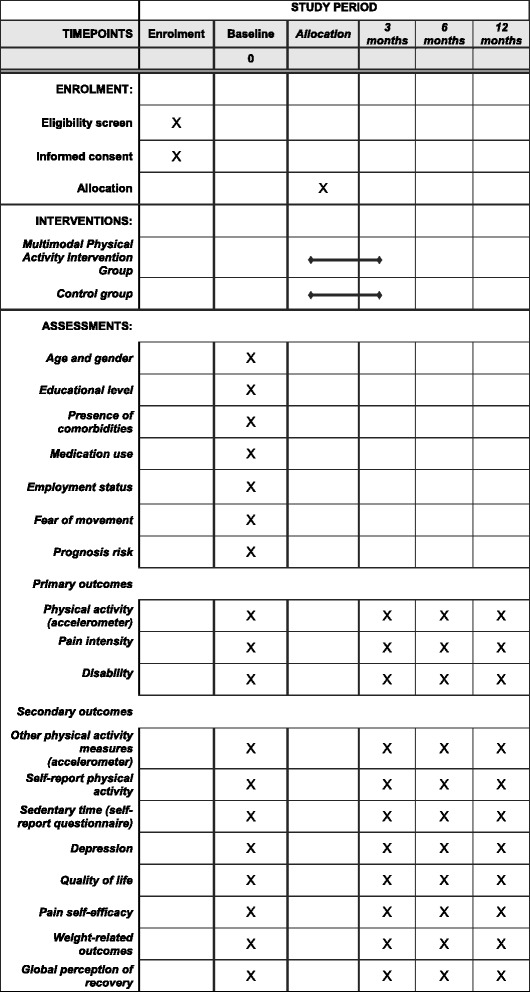
Details of the schedule of enrolment, interventions and assessments according to Standard Protocol Items: Recommendations for Intervention Trials (SPIRIT) Diagram

This study will be a parallel randomised controlled trial (RCT) conducted at two outpatient physical therapy clinics in Presidente Prudente, Brazil. This protocol conforms to the Consolidated Standard of Reporting Trials (CONSORT) Statement and is registered at ClinicalTrials.gov (NCT03200509). Figure 1 shows the study design. The Standard Protocol Items: Recommendations for Intervention Trials (SPIRIT) Checklist is provided in Additional file 1 and the SPIRIT Diagram is included in the Fig. 2.

### Sample size

A sample size calculation was performed based on an objective measure of physical activity level, i.e. counts per min, derived from an accelerometer. A total of 160 participants (80 patients per group) will be required to detect a 20% between-group difference in physical activity levels (mean difference between groups of 59.2 counts per min, a standard deviation of 111.6 counts per min) with a power of 0.80, alpha of 0.05 accounting for a 15% loss to follow-up. The counts per min parameters used in the sample size calculation are from a previous study conducted with a similar population [22]. The total of 160 participants is enough to detect a between-group difference of 1 point (standard deviation (SD) = 1.84) in the numerical pain rating scale and of 4 points (SD = 4.9) in the Roland Morris Disability Questionnaire (RMDQ) with a power of 80%, an alpha of 0.05 and 15% dropout as reported in a previous trial with this population [23].

### Participants

Participants with nonspecific LBP seeking care at two outpatient physiotherapy clinics will be invited to participate in this trial. The recruitment will start in August 2017 and is expected to finish in December 2018 with final data collection (12-month follow-up) in December 2019. Patients with chronic, nonspecific LBP, defined as pain and discomfort localised below the costal margin and above the inferior gluteal folds, with or without leg pain and of at least 3 months’ duration, will be included if they are aged between 18 and 60 years. Participants who have serious spinal pathology (e.g. tumours, fractures and inflammatory diseases), nerve root compromise (i.e. at least two of the following signs: weakness, reflex change, or sensory loss associated with the same spinal nerve), spinal surgery, pregnancy, illiteracy, insufficient understanding of the Portuguese language, cardiorespiratory diseases, fibromyalgia or any other musculoskeletal condition that may affect activity and movement will be excluded.

A screening assessment to check the eligibility criteria will be undertaken by a trained physiotherapist. Then, participants who meet the eligibility criteria will be provided with verbal and written information about the purpose of the study. Participants who agree to participate in the study will be asked to give written informed consent before the baseline assessments.

### Procedures

After baseline assessment, the participants will be randomised to the intervention or the control group. Randomisation will be undertaken using a random sequence of numbers generated by computer software (Microsoft Excel). This sequence will be generated and inserted in opaque and sealed envelopes by a research assistant. After baseline assessment, the treating physiotherapist will open the envelope, reveal the treatment allocation and deliver the interventions. The treating physiotherapist will not be blinded to group allocation due to the nature of the intervention. Trained assessors blinded to group allocation will be responsible for outcome measurement.

### Interventions

The participants from both groups will receive a group exercise programme, including a combination of general, stabilisation, strengthening and resistance exercises. The group exercise programme will be led by a physiotherapist with at least 2 years’ clinical experience and consists of a 45-min group session with up to 10 people, delivered twice a week for 3 months. See Table 1 for details of the intervention which accords to the Description of the intervention using the Template for Intervention Description and Replication (TIDieR) Checklist.

**Table 1:** Description of the intervention using the Template for Intervention Description and Replication (TIDieR) Checklist

1. Brief name	The Physical Activity for Back Pain (PAyBack) trial
2. Why?	Low back pain (LBP) is one of the most disabling conditions imposing an enormous economic burden to society and individuals. Although physical activity and exercise are effective in reducing pain and disability in patients with chronic LBP, these improvements are at best moderate and not sustained over the long term. Furthermore, physical activity-based interventions are not effective for increasing physical activity levels of patients with chronic musculoskeletal pain. We would argue that current interventions are not designed to change patients’ behaviour toward an active lifestyle
3. What materials?	Participants allocated to the intervention group will receive the activity monitor *Fitbit Flex*. For the purpose of the current study, we will blind participants to the feedback lights by covering all wristbands with heat-shrink tubing. Participants allocated to the control group will receive a *Fitbit Flex* wristband without the accelerometer containing a material that mimics the weight of the accelerometer and the heat shrink tubing to cover the wristband as in the intervention group
4. What procedures?	The participants from both groups will receive a group-based exercise programme, including a combination of general, stabilisation, aerobic, strengthening and resistance exercises: *Multimodal physical activity intervention group* - Health coaching sessions to identify facilitators and barriers to physical activity participation and to assist participants to achieve their physical activity goals by providing ongoing education and support. An individualised and realistic physical activity plan will be developed for each participant, considering the presence of potential cardiovascular risk factors and participants’ current activity level. In addition, during the health coaching sessions, the physiotherapist will educate patients on the benefits of staying active and will help them to recognise and control, if relevant, pain-related fear, catastrophising and negative thoughts and emotions about pain - *Fitbit Flex* to give feedback on the amount of daily physical activity achieved, used by the participants as a motivational tool and to provide feedback during the health coaching sessions *Control group* - Sham coaching giving participants the opportunity to talk about their LBP or other problems, but without providing any therapeutic advice - Sham *Fitbit* providing the same information given to the intervention group
5. Who provided?	A physiotherapist who attended a health coaching course, obtained practice experience from experienced health coaches and trained to use active listening techniques
6. How?	Supervised exercises will be delivered in group exercises sessions. The health coaching and sham coaching sessions will be delivered on an individual basis via face-to-face and telephone contact
7. Where?	The trial will be conducted at two outpatient physiotherapy clinics in Presidente Prudente, Sao Paulo, Brazil.
8. When and how much?	The group exercise programme will be led by a physiotherapist with at least 2 years’ clinical experience and will consist of 45-min group sessions with up to 10 people, twice a week for 3 months For health coaching, a total of 12 health coaching sessions, each session lasting for 30–60 min, will be provided over a 3-month period. The intervention will be delivered during one face-to-face visit each week for the first 6 weeks and then during one telephone contact each week for the last 6 weeks. The participants will be encouraged to wear the *Fitbit* during all waking hours for the 3-month intervention period For the control group, the sham health coaching will consist of one weekly session (first 6 weeks being a face-to-face session and the last 6 weeks by telephone contact), each session lasting for 30–60 min, over a 3-month period. In addition, the participants will receive the sham *Fitbit* and be encouraged to wear it during waking hours for the 3-month study period
9. Tailoring	For the intervention group, the individualised activity plan will be delivered considering the presence of potential cardiovascular risk factors and participants’ current activity level. In addition, the physical activity goals will focus on attempting to meet the physical activity recommendations of at least 150 min of moderate-intensity or 75 min of vigorous-intensity physical activity throughout the week or walking between 7000 to 10,000 steps per day

#### Multimodal physical activity intervention group

Participants randomised to the intervention group will receive, in addition to the exercise programme, a health coaching programme together with an activity monitor. The health coaching will be provided by a physiotherapist who has attended a health coaching course (http://www.wellnesscoachingaustralia.com.au) and has been mentored by experienced health coaches participating in ongoing trials [24, 25]. The purpose of the health coaching is to identify facilitators and barriers to physical activity participation and to assist participants to achieve their physical activity goals by providing ongoing education and support. The physical activity goals will focus on reducing time spent in sedentary activities as well as attempting to meet the physical activity recommendations of at least 150 min of moderate-intensity or 75 min of vigorous-intensity physical activity throughout the week [26] or walking between 7000 to 10,000 steps to per day [27, 28]. An individualised and realistic physical activity plan will be developed during the first health coaching session between the physiotherapist and the participant considering the presence of potential cardiovascular risk factors and participants’ current physical activity. During the health coaching session, the physiotherapist will educate patients on the benefits of staying active and will help them to recognise and control, if relevant, pain-related fear, catastrophising and negative thoughts and emotions about pain [29]. A total of 12 health coaching sessions, each session lasting for 30–60 min, will be provided over a 3-month period. The intervention will be delivered during one face-to-face visit each week for the first 6 weeks and then during one telephone contact each week for the last 6 weeks.

**Fig. 3:**
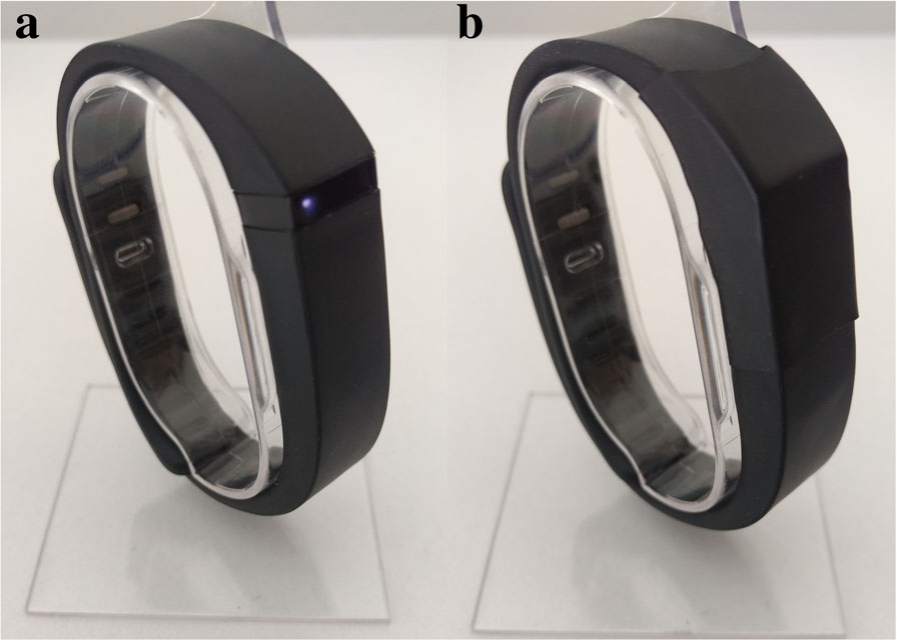
The *Fitbit Flex*. **a** the original *Fitbit Flex*. **b** the *Fitbit Flex* with heat-shrink tubing covering the indicator lights

The activity monitor, *Fitbit Flex* (*Fitbit* Inc., San Francisco, CA, USA), will be given to all participants in the intervention group. The *Fitbit Flex* is a small (140–176 mm) three-axis accelerometer inserted in a wristband designed to give feedback on the amount of daily physical activity achieved. Participants will be encouraged to wear the activity monitor during waking hours. The *Fitbit Flex* has five indicator lights that flash various patterns depending on what the tracker is doing. For the purpose of the current study, we will blind the participants to the lights by covering all wristbands with heat-shrink tubing (Fig. 3). The activity monitor data will be uploaded while the participants are attending the group exercise classes and the physiotherapist will use these data to provide feedback on the amount of physical activity during the health coaching sessions. The activity monitors will be charged while the physical activity data are being uploaded.

#### Control group

The participants allocated to the control group will receive, in addition to the exercise programme, sham health coaching and a sham activity monitor. Sham health coaching will consist of one weekly session (the first 6 weeks will be a face-to-face session and the last 6 weeks will be telephone contacts), each session lasting for 30–60 min, over a 3-month period, based on a ‘reflective, non-directive approach’ described previously elsewhere [30]. The physiotherapist will be trained to use active listening techniques, giving participants the opportunity to talk about their LBP or other problems. In response, the treating physiotherapist will reply in a warm and empathic manner, with a special interest in the report, but without providing any therapeutic advice. This strategy is designed to control for time with a physiotherapist and the therapeutic alliance that occurs within a consultation. A previous study found that this approach is credible which maximises participant blinding [30].

All participants in the control group will be given a sham activity monitor, which will consist of a *Fitbit Flex* wristband without the accelerometer. A material that mimics the weight of the accelerometer will be inserted in the wristband and heat-shrink tubing will be used to cover the wristband as in the intervention group. The same information given to the intervention group about the activity monitor will be provided to the participants in the control group. Nevertheless, the participants will be informed that the treating physiotherapist does not have access to the *Fitbit* data for the control group. Participants in the control group will give their sham *Fitbit* to the physiotherapist before each exercises class and will be told that this procedure is needed to charge the *Fitbit*.

### Outcomes

The measures will be collected using REDCap (Research Electronic Data Capture) [31] hosted at Sao Paulo State University. Data will be collected and stored in spreadsheets by assessors blinded to group allocation. At baseline assessment, the physiotherapist assessors will collect data on participant characteristics (i.e. age, gender, educational level, presence of comorbidities, medication use and employment status). To characterise the study participants, information on fear of movement measured with the Tampa Scale for Kinesiophobia (TSK) [32] and prognosis risk measured with the Örebro Musculoskeletal Pain Screening Questionnaire (ÖMPSQ) [33] will be collected at baseline. Outcome measures will be assessed at baseline, post intervention (i.e. 3 months after randomisation) and at 6 and 12-month follow-ups. Details on the primary and secondary outcomes are reported below:

#### Primary outcomes

- Physical activity – counts per min is the main objective measure of physical activity and will be measured with the Actigraph GT3X (ActiGraph, LLC, Pensacola, FL, USA). The Actigraph GT3X is a non-invasive, small (4.6 × 3.3 × 1.5 cm) triaxial accelerometer worn above the right hip for seven consecutive days during waking hours. Acceleration data will be sampled at 30 Hz and analysed at 60-s epochs. A complete data set for each patient will be defined as having at least 10 h/day of monitored wear over at least 5 days [34]. Non-wear periods will be defined as time intervals of at least 60 consecutives min of zero counts, with an activity interruption allowance of 0 to 100 counts/min lasting a maximum of two consecutive days [35]. These parameters have been used in previous studies from our group [22, 36]. Counts per min will be calculated by dividing the sum of activity counts of the vertical axis by the number of valid days
- Disability – the Roland Morris Disability Questionnaire (RMDQ) [37] will be used to measure disability. The RMDQ consists of 24 yes-or-no questions with total scores ranging from 0 to 24 and higher scores indicating greater disability
- Pain intensity – Numerical Rating Scale for pain assessment (NRS) will be used to measure pain intensity. The NRS evaluates the pain intensity in the last week through an 11-point-scale from 0 to 10 with higher scores indicating greater pain intensities

#### Secondary outcomes

- Other objective measures of physical activity, such as time spent in light and moderate-vigorous physical activity, number of steps and time spent in sedentary behaviour, will be considered secondary outcomes. These data will be collected with the same procedure reported for counts per min. Sedentary time will be defined as values less than 100 count/min, light physical activity will be defined as values between 100 and 2019 counts/min and moderate-vigorous physical activity will be defined as values greater than 2020 counts/min [34]
- Self-reported physical activity level will be measured with the Baecke Habitual Physical Activity Questionnaire [22]. This questionnaire consists of 16 items covering three dimensions: leisure-time and locomotive physical activities; leisure-time physical exercises; and occupational physical activities. The total score ranges from 3 to 15, with higher scores indicating higher physical activity levels
- Self-reported sedentary behaviour will be measured with questions about the time spent in sedentary behaviour across five different domains in a usual weekday [38]: workplace, commuting, school/university, watching TV, and computer use. Before quantifying the time spent in each sedentary activity, the participants will be asked if they are exposed, or not, to each behaviour. We will use the sum of the domains as well as the time spent in each sedentary behaviour
- Depression will be assessed with The Center for Epidemiological Studies – Depression (CES-D) scale [39]. The CES-D scale assesses the frequency of depression symptoms in the last week with 20 questions. Total scores range from 0 to 60 and higher scores indicate higher depression levels
- Quality of life will be assessed using the EuroQol Visual Analogue Scale (EQ-VAS) [40]. In the EQ-VAS, respondents report their perceived health status with a grade ranging from 0 (the worst possible health status) to 100 (the best health status)
- The Pain Self-Efficacy Questionnaire (PSEQ) [41] will be used to assess pain self-efficacy. The PSEQ contains 10 items with scores ranging from 0 to 6. Final scores range from 0 to 60 with higher scores indicating more confidence to perform an activity despite the pain
- Weight-related outcomes – Body Mass Index and waist-to-hip circumference will be used to measure weight-related outcomes. Body Mass Index will be calculated based on weight and height measured by a digital scale and an stadiometer and waist and hip circumference using an anthropometric measuring tape. Waist circumference will be measured midway between the 10th rib and the top of the iliac crest and hip circumference as the widest part over the buttocks to the nearest 0.5 cm

At the post-intervention assessment, we will also collect the subjective perception of recovery with the Global Perceived Effect Scale (GPES) [42] that ranges from − 5 to + 5 with higher scores indicating higher recovery from the condition. In addition, the participants will rate the credibility of the treatments using a scale (Appendix 1) consisting of four questions on a 7-point scale with scores ranging from 0 (not confident or not logical) to 6 (absolutely confident or very logical) [30].

### Data analysis

Continuous variables will be reported using mean (standard deviation) or median (interquartile range), depending on the data distribution, and dichotomous and categorical variables will be reported using frequencies (proportion). All data will be analysed following intention-to-treat principles. The difference between groups will be analysed with linear mixed models using fixed effects for group, time and group-versus-time interaction and random intercepts for individuals to account for the dependence of repeated measures. Statistical significance will be set at 0.05. We will report the number of participants with missing scores for each outcome. The statistical software SPSS V.20.0 (IBM corporation, Somers, NY, USA) will be used for data analysis. Planned subgroup analyses will investigate differences in effects of the intervention by physical activity levels at baseline assessment and pain self-efficacy. In addition, we will also conduct secondary analyses to investigate the treatment effects considering the adherence to treatment using a complier average causal effect (CACE) approach.

## Discussion

This trial is significant as it will be the first study to investigate the efficacy of a multimodal intervention designed to increase physical activity levels compared to a control intervention in patients with chronic LBP. We will test whether the proposed intervention reduces pain and disability, but at the same time increases physical activity levels. More importantly, the long-term follow-up of 6 and 12 months will allow us to investigate whether these effects are sustained over time after the completion of the intervention.

For the physiotherapy field, this study will provide important elucidation about physiotherapists delivering interventions to promote physical activity. Indeed, the physiotherapists are in a privileged position to promote physical activity and, consequently, to prevent non-communicable diseases, improve biomedical outcomes and the lifelong health of patients [43]. Although previous studies have shown that physiotherapists are not confident to change patients’ behaviour [44], this type of strategy should be viewed as a professional responsibility considering patients’ health.

A strength of this trial is the use of a sham control treatment to determine the efficacy of an intervention to promote physical activity. In addition, our trial is designed to conform to SPIRIT and CONSORT guidelines and will be sufficiently powered to detect a difference in the primary outcomes. The limitation of this study is the lack of blinding of the treating physiotherapists due to the nature of the intervention.

### Trial status

The recruitment started in August 2017 and is expected to finish in December 2018 with final data collection (12-month follow-up) in December 2019.

## Additional file

Additional file 1: Standard Protocol Items: Recommendations for Intervention Trials (SPIRIT) Checklist. (PDF 151 kb)

## Abbreviations

CES-D: The Center for Epidemiological Studies – Depression
CONSORT: Consolidated Standard of Reporting Trials
EQ-VAS: EuroQol Visual Analogue Scale
GPES: Global Perceived Effect Scale
LBP: Low back pain
NRS: Numerical Rating Scale for pain assessment
ÖMPSQ: Örebro Musculoskeletal Pain Screening Questionnaire
PAyBACK: Physical Activity for Back Pain
PSEQ: Pain Self-Efficacy Questionnaire
RCT: Randomised controlled trial
RMDQ: Roland Morris Disability Questionnaire
SPIRIT: Standard Protocol Items: Recommendations for Intervention Trials
TSK: Tampa Scale for Kinesiophobia
